# The association between tocilizumab therapy and the development of thrombosis in critically ill patients with COVID-19: a multicenter, cohort study

**DOI:** 10.1038/s41598-024-53087-z

**Published:** 2024-02-06

**Authors:** Ohoud Aljuhani, Khalid Al Sulaiman, Ghazwa B. Korayem, Ali F. Altebainawi, Samiah Alsohimi, Rahaf Alqahtani, Saeedah Alfaifi, Aisha Alharbi, Azzah AlKhayrat, Ahmed Hattan, Meshal Albassam, Omar A. Almohammed, Atheer Alkeraidees, Dhay A. Alonazi, Weam F. Alsalman, Ghaliah Aldamegh, Rasha Alshahrani, Ramesh Vishwakarma

**Affiliations:** 1 Department of Pharmacy Practice, Faculty of Pharmacy, King Abdulaziz University, Jeddah, Saudi Arabia; 2 Pharmaceutical Care Department, King Abdulaziz Medical City, Riyadh, Saudi Arabia; 3 College of Pharmacy, King Saud bin Abdulaziz University for Health Sciences, Riyadh, Saudi Arabia; 4 King Abdullah International Medical Research Center, Riyadh, Saudi Arabia; 5 Department of Pharmacy Practice, College of Pharmacy, Princess Nourah bint Abdulrahman University, Riyadh, Saudi Arabia; 6 Pharmaceutical Care Services, King Salman Specialist Hospital, Hail Health Cluster, Hail, Saudi Arabia; 7 Pharmaceutical Care Department, King Fahad Armed Forces Hospital, Jeddah, Saudi Arabia; 8 Pharmaceutical Care Services, King Abdulaziz University Hospital, Jeddah, Saudi Arabia; 9 Pharmaceutical Care Department, Dallah Hospital, Riydah, Saudi Arabia; 10 Pharmaceutical Care Department, King Abdulaziz Medical City, Jeddah, Saudi Arabia; 11 Pharmaceutical Care Services, Prince Sultan Military Medical City, Riyadh, Saudi Arabia; 12 Pharmaceutical Care Services, King Abdullah bin Abdulaziz University Hospital, Riyadh, Saudi Arabia; 13 Department of Internal Medicine & Critical Care, King Abdullah Bin Abdulaziz University Hospital, Riyadh, Saudi Arabia; 14 College of Pharmacy, King Saud University, Riyadh, Saudi Arabia; 15 Pharmaceutical Care Department, King Saud Medical City, Riyadh, Saudi Arabia; 16 Norwich clinical trial unit, Norwich Medical School, University of East Anglia, Norwich, UK; 17 King Abdulaziz Medical City (KAMC)-Ministry of National Guard Health Affairs (MNGHA), King Abdullah International Medical Research Center, King Saud bin Abdulaziz University for Health Sciences, PO Box 22490, 11426 Riyadh, Saudi Arabia

**Keywords:** Drug therapy, Viral infection

## Abstract

The use of tocilizumab for the management of COVID-19 emerged since it modulates inflammatory markers by blocking interleukin 6 receptors. Concerns regarding higher thrombosis risk while using tocilizumab were raised in the literature. The aim of this study is to investigate the association between tocilizumab therapy and the development of thromboembolic events in critically ill COVID-19 patients. A propensity score-matched, multicenter cohort study for critically ill adult patients with COVID-19. Eligible patients admitted to ICU between March 2020 and July 2021 were categorized into two sub-cohorts based on tocilizumab use within 24 h of ICU admission. The primary endpoint was to assess the incidence of all thrombosis cases during ICU stay. The secondary endpoints were 30-day mortality, in-hospital mortality, and the highest coagulation parameters follow-up (i.e., D-dimer, Fibrinogen) during the stay. Propensity score matching (1:2 ratio) was based on nine matching covariates. Among a total of 867 eligible patients, 453 patients were matched (1:2 ratio) using propensity scores. The thrombosis events were not statistically different between the two groups in crude analysis (6.8% vs. 7.7%; p-value = 0.71) and regression analysis [OR 0.83, 95% CI (0.385, 1.786)]. Peak D-dimer levels did not change significantly when the patient received tocilizumab (beta coefficient (95% CI): 0.19 (− 0.08, 0.47)), while there was a significant reduction in fibrinogen levels during ICU stay (beta coefficient (95% CI): − 0.15 (− 0.28, − 0.02)). On the other hand, the 30-day and in-hospital mortality were significantly lower in tocilizumab-treated patients (HR 0.57, 95% CI (0.37, 0.87), [HR 0.67, 95% CI (0.46, 0.98), respectively). The use of tocilizumab in critically ill patients with COVID-19 was not associated with higher thrombosis events or peak D-dimer levels. On the other hand, fibrinogen levels, 30-day and in-hospital mortality were significantly lower in the tocilizumab group. Further randomized controlled trials are needed to confirm our findings.

## Introduction

The Coronavirus disease 2019 (COVID-19) pandemic has affected more than six hundred million cases and killed more than six million people globally^1^. Approximately 20% of COVID-19 cases progress to the moderate-to-severe disease stage^2^. These patients need hospitalization and oxygen support, and up to 5% of them may need admission to the intensive care unit (ICU)^2^. These patients present with severe inflammatory response also referred to as a “cytokine storm,” which is one of the hallmarks of severe COVID-19 case; this condition is distinguished by the elevated levels of inflammatory cytokines such as interleukin 1 (IL-1), IL-6, tumor necrosis factor, and other cytokines^3^. The cytokine storm is associated with several life-threatening complications, including coagulopathy and failure of multiple organs^4^. Thus, several treatment modalities, such as immunomodulator drugs like Tocilizumab (TCZ) have been proposed and investigated to manage severe cases of COVID-19^5^.

The available evidence supports the mortality benefit of using TCZ in severe cases of COVID-19^6–10^. Despite the mortality benefit of using TCZ in patients with COVID-19, safety concerns are still associated with its use^11–16^. Moreover, there were concerns about increased d-dimer and mortality secondary to thrombosis risk with tocilizumab administration^17^. The association between IL-6 and venous thromboembolism is complex. Several experimental studies including non-COVID-19 patients reported that IL-6 elevation can increase the risk of deep vein thrombosis through various mechanisms^18,19^.

The IL-6 elevation was greater in patients who died and were not on therapeutic anticoagulation^17^. In addition, a case series reported four cases of venous thromboembolism (VTE) and one case of an arterial thrombotic event in which TCZ has been used as an adjunct therapy to the standard of care to treat patients with SARS-CoV-2 despite using prophylactic anticoagulants^20^.

In contrast, a systematic review and meta-analysis that evaluated the efficacy and safety of TCZ in the treatment of COVID-19 illustrated no significant difference in pulmonary thrombosis rates between TCZ and the control group^21^. An additional systematic review and meta-analysis demonstrated a tendency toward fewer VTEs in individuals using IL-6 inhibitors. However, this rate was not statistically significant^22^. Therefore, the relationship between the use of IL-6 antagonists and the occurrence of thromboembolic events in patients with COVID-19 remains unclear^17,22^. In light of this, the purpose of this study is to investigate the association between TCZ therapy and the development of thromboembolic events in critically ill patients with COVID-19.

## Methods

### Study design

This study is part of the Saudi Critical Care Pharmacy Research (SCAPE) platform, which has undertaken various studies to assess the safety and effectiveness of a range of treatments and therapies for patients in critical condition (Saudi Critical Care Pharmacy Research (SCAPE), 2023). This is a multicenter retrospective cohort study included five centers located in various regions in Saudi Arabia between March 1, 2020, and July 31, 2021. Eligible critically ill COVID-19 patients during the study period were categorized to two sub-cohorts based on early Tocilizumab use within 24 h of ICU admission (Tocilizumab versus Control). Control was defined as patients who did not receive Tocilizumab during ICU stay. Reverse transcriptase-polymerase chain reaction (RT‒PCR) was used for COVID-19 diagnosis confirmation. All included patients were followed until they were discharged from the hospital or died during the hospital stay. The study was conducted in accordance with the World Medical Association Declaration of Helsinki—Ethical Principles for Medical Research Involving Human Subjects (adopted 1964; updated 2013), national ethical regulations, and local institutional guidance of study centers. The study was approved by King Abdullah International Medical Research Center (KAIMRC) institutional review board in February 2021 (Ref.# NRC21R.024.01). The IRB committee waived informed consent from the study patients due to the retrospective observational nature of the study.

### Settings

We included patients from five centers in Saudi Arabia. Centers selected based on research feasibility, data availability and geographical location. Data obtained from five hospitals, two of which were in Riyadh city, two in Jeddah and one hospital in Hail city. Participated institutions were King Abdulaziz Medical City (Riyadh, Jeddah), King Abdulaziz University Hospital (Jeddah), King Abdullah bin Abdulaziz University Hospital (KAAUH) (Riyadh), and King Salman Specialist Hospital (Hail). The primary site for this multicenter study was King Abdulaziz Medical City (KAMC- Riyadh), a tertiary care center. The initial IRB approval originated from King Abdulaziz Medical City (Riyadh).

### Study participants

**Figure 1:**
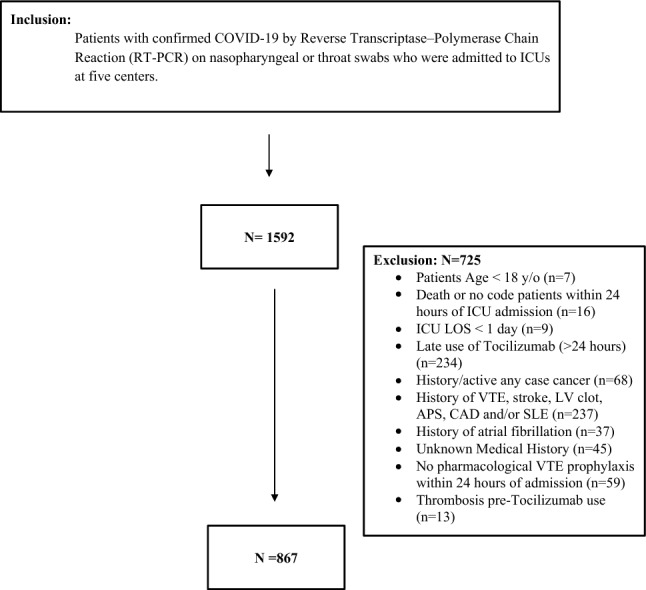
Flow diagram showing patients recruited with COVID-19. COVID-19, Coronavirus disease; ICU, intensive care unit; LOS, length of stay.

Patients who were admitted to ICUs between March 2020 and July 2021 in participating hospitals were screened for inclusion. Patients included if they were adult with age of ≥ 18 years and confirmed COVID-19 infection with polymerase chain reaction (PCR) testing. To minimize confounding factors, patients who had history of thrombosis, known to have risk of thrombosis (i.e. CAD, SLE, APS), atrial fibrillation prior to ICU admission, had history/active cancer, labled as DNR, deceased with 24 h of ICU admission, delayed tocilizumab start > 24 h of ICU admission, or omitted from chemo-prophylactic anticoagulation were excluded (Fig. 1).

### Outcomes

This study aims to evaluate the association between Tocilizumab use and thrombosis in critically ill patients with COVID-19. The primary endpoint was to assess the incidence of all thrombosis cases during ICU stay. The secondary endpoints were 30-day mortality, in-hospital mortality, and the coagulation parameters follow-up (i.e., D-dimer, Fibrinogen) during the stay.

### Outcomes definition

- All cases of thrombosis (arterial and venous) were confirmed with radiological studies including ultrasound and computed tomography scans and/or chart documentation (i.e., myocardial infarction (MI), ischemic stroke, pulmonary embolism, deep vein thrombosis).
- The 30-day mortality was defined as a death occurring for any cause within 30 days of the admission date during hospital stay; patients who were discharged from the hospital alive before 30 days were presumed to be survivors.
- In-hospital mortality is defined as death occurring for any cause date the hospital stay.

### Data collection

The database was created utilizing Research Electronic Data Capture (REDCap®) platform hosted by King Abdullah International Medical Research Center (KAIMRC). All collaborators were trained on the use of the REDCap® as well as data variables. Demographic variables were collected in addition to clinical ICU variables such as APACHE and SOFA scores, mechanical ventilation needs, and settings. Moreover, comprehensive laboratory variables, radiological findings, comorbidities, and the use of DVT prophylaxis and inflammatory markers were also captured for eligible patients. Lastly, the peak levels of D-dimer and fibrinogen during ICU stay were also collected to assess the effect of tocilizumab on these markers.

### Sample size

Expecting a 12% prevalence of thrombosis among hospitalized patients, we anticipate a reduction to 3.7% in the tocilizumab group^25,26^. Using this assumption, we determined a sample size of 446 to achieve a statistical power of 90% and maintain an alpha error below 5%.

### Statistical analysis

Continuous variables were reported using mean and standard deviation (SD), or median with lower and upper quartile (Q1, Q3), and categorical variables as number (percentage) when appropriate. We compared two study groups by using either Chi-square or Fisher exact test for categorical variables. Continues variables were compared using Student t-test to compare (normally distributed continuous data) and Mann–Whitney U test to compare (non-normally distributed continuous variables). In addition, statistical test (the Shapiro–Wilk test) and graphical representation (i.e., histograms and Q–Q plots), were evaluated the normality assumptions for all numerical variables.

Multivariable logistic and negative binomial regression analysis were used for binomial and continuous outcomes included in this study, respectively. Multivariable Cox proportional hazards regression analyses were generated to determine the 30-day and in-hospital mortality. Regression analysis was made by considering the PS score as one of the covariates in the model. The odds ratios (ORs), estimates, or hazard ratios (HRs) with 95% confidence intervals (CIs) were reported for the associations. We assessed the model fit using the Hosmer–Lemeshow goodness-of-fit test.

In the propensity score-matched analysis, we selected nine matching covariates, including age, body mass index (BMI), APACHE II score, baseline INR, early use of dexamethasone, acute kidney injury at admission, chronic kidney disease, heart failure and liver disease as a comorbid condition. Those factors were selected for their possible association with the study outcomes. A greedy nearest-neighbor matching method was used in which one patient who received Tocilizumab was matched with two patients in the control group (1:2 ratio). This eventually produces the smallest within-pair difference among all available pairs with treated patients. These patients were matched only if the difference in the logits of the propensity scores for pairs of patients from the two groups was less than or equal to 0.1 times the pooled estimate of the standard deviation. We used a caliper width of 0.1 and the standardized mean difference (SMD) was used to examine the degree of PSM. Less than 0.1 was considered an acceptable threshold. The primary outcome was further verified using inverse probability of treatment weighted (IPTW), which was created using the estimated propensity scores as weights. The details of propensity score matching results are included in the appendix. We considered a p value of < 0.05 statistically significant and used SAS version 9.4 for all statistical analyses.

### Ethics approval and consent to participate

The study was approved in February 2021 by King Abdullah International Medical Research Center (KAIMRC)—Institutional Review Board (IRB), Riyadh, Saudi Arabia (Ref.# NRC21R.024.01). Informed consent was waived KAIMRC-IRB due to the retrospective nature of the study. Participants’ confidentiality was strictly observed throughout the study by using anonymous unique serial number for each subject and restricting data only to the investigators.

## Results

### Demographic and clinical characteristics

Among 867 patients who met the inclusion criteria (Fig. 1), Tocilizumab was given to 256 patients during the study period. A total of 453 patients were included in the study after being matched by propensity score (1:2 ratio). In the overall cohort and before PS matching, the majority of patients were males (61.1%) with an average age of 61.2. (± 14.99). Diabetes mellitus was the most common comorbid condition in the whole cohort (58.4%), followed by hypertension (55.4%) and dyslipidemia (21.1%). Before PS matching, patients who received early Tocilizumab were younger, had a lower APACHE II score, serum creatinine, baseline AKI, total WBCs, INR, aPTT, total bilirubin, D-dimer, and lower PaO2/FiO2. In contrast, the control group had a lower BMI and early use of Dexamethasone within 24 h of ICU admission. Post PS score matching, both groups had comparable baseline variables except for baseline INR, C-reactive protein, and ferritin levels. The intensity of pharmacological DVT prophylaxis was similar between the two groups after PS matching. Table 1 summarizes all the baseline characteristics before and after PS matching in patients who received Tocilizumab compared with the control.

**Table 1:** Baseline characteristics of patients admitted to the ICU.

	Before propensity score (PS)				After propensity score (PS)			
	Overall (N = 867)	Control (N = 611)	Early tocilizumab (N = 256)	p-value	Overall (N = 453)	Control (N = 302)	Early tocilizumab (N = 151)	p-value
Age (Years), mean (SD)	61.2 (14.99)	61.9 (15.35)	59.3 (13.97)	0.0171^	59.1 (14.48)	59.4 (14.96)	58.4 (13.50)	0.4804*
Gender—male, n (%)	511 (61.1)	365 (62.1)	146 (58.9)	0.3854^^	289 (64.8)	190 (64.0)	99 (66.4)	0.6065^^
BMI, mean (SD)	31.7 (9.75)	31.0 (8.64)	33.4 (11.85)	0.0008^	31.9 (9.25)	31.6 (9.52)	32.5 (8.68)	0.2253^
APACHE II score, median (Q1, Q3)	12.0 (8.00, 19.00)	13.0 (8.00, 20.00)	12.0 (8.00, 15.00)	0.0082^	11.0 (7.00, 16.00)	11.0 (7.00, 17.00)	11.0 (8.00, 15.00)	0.8736^
SOFA score, median (Q1, Q3)	4.0 (2.00, 7.00)	4.0 (2.00, 7.00)	4.0 (2.50, 6.00)	0.1268^	4.0 (2.00, 6.00)	4.0 (2.00, 6.00)	4.0 (2.00, 6.00)	0.8840^
Vasoactive-Inotropic score, mean (SD)	5.8 (40.5)	7.1 (45.0)	2.8 (27.2)	0.07^	4.3 (33.2)	4.1 (23.4)	4.5 (34.9)	0.64^
Early use of dexamethasone within 24 h of admission, n (%)	564 (66.6)	355 (59.5)	209 (83.6)	< 0.0001^^	346 (77.4)	228 (76.5)	118 (79.2)	0.5223^^
Serum creatinine (mmol/L) at admission, median (Q1, Q3)	82.0 (66.00, 120.00)	85.3 (66.00, 127.00)	77.0 (64.00, 103.00)	0.0217^	78.0 (65.00, 105.00)	78.0 (65.00, 109.00)	76.0 (63.00, 101.20)	0.6572^
Acute Kidney Injury (AKI) within 24 h of ICU admission, n (%)	207 (24.8)	165 (28.1)	42 (16.9)	0.0007^^	93 (20.8)	65 (21.8)	28 (18.8)	0.4584^^
Mechanical ventilation within 24 h of ICU admission, n (%)	581 (69.1)	415 (69.9)	166 (67.2)	0.4473^^	304 (68.0)	201 (67.4)	103 (69.1)	0.7200^^
Lowest MAP at admission, median (Q1, Q3)	73.0 (63.00, 83.00)	73.0 (63.00, 83.67)	73.0 (65.00, 80.50)	0.7683^	75.0 (65.00, 83.00)	75.0 (64.00, 84.00)	75.0 (66.50, 81.00)	0.6671^
Lactic acid baseline, median (Q1, Q3)	1.7 (1.27, 2.35)	1.7 (1.24, 2.38)	1.7 (1.32, 2.34)	0.8343^	1.6 (1.29, 2.17)	1.6 (1.30, 2.17)	1.6 (1.28, 2.18)	0.8539^
Platelets count baseline, median (Q1, Q3)	250.0 (193.00, 319.00)	248.0 (190.00, 320.00)	253.0 (201.50, 317.00)	0.4271^	253.0 (198.00, 318.00)	249.0 (191.00, 315.00)	256.0 (204.00, 323.00)	0.1778^
Total WBC baseline, median (Q1, Q3)	9.0 (6.46, 12.50)	9.4 (6.57, 12.60)	8.3 (6.21, 11.65)	0.0288^	8.7 (6.27, 12.00)	8.9 (6.29, 12.00)	8.2 (6.22, 11.40)	0.6567^
International normalized ratio (INR), median (Q1, Q3)	1.1 (1.00, 1.15)	1.1 (1.01, 1.19)	1.0 (0.97, 1.10)	0.0001*	1.1 (1.00, 1.13)	1.1 (1.00, 1.14)	1.1 (0.99, 1.11)	0.0322^
Activated partial thromboplastin time (aPTT) baseline, median (Q1, Q3)	29.3 (26.10, 32.80)	29.6 (26.40, 33.10)	28.3 (26.00, 31.60)	0.0173^	29.3 (26.10, 32.60)	29.3 (26.25, 32.80)	29.1 (26.10, 32.00)	0.5399^
Total bilirubin, median (Q1, Q3)	9.0 (6.30, 13.00)	9.1 (6.70, 13.50)	8.6 (6.00, 12.00)	0.0364^	8.9 (6.60, 12.90)	8.5 (6.45, 12.80)	9.2 (6.80, 13.10)	0.2629^
Alanine aminotransferase (ALT), median (Q1, Q3)	36.0 (24.00, 58.00)	36.0 (24.00, 60.00)	38.0 (25.00, 56.00)	0.6974^	37.0 (24.00, 60.00)	36.0 (24.00, 61.00)	38.0 (24.00, 56.00)	0.8013^
Aspartate aminotransferase (AST), median (Q1, Q3)	50.0 (33.00, 75.00)	49.5 (32.00, 75.00)	50.0 (36.00, 74.00)	0.4411^	48.0 (31.00, 72.00)	47.0 (31.00, 72.00)	49.0 (35.00, 70.00)	0.8091^
Albumin baseline, median (Q1, Q3)	33.0 (29.00, 36.00)	32.8 (28.00, 36.00)	33.0 (30.00, 36.00)	0.1037^	33.0 (29.00, 36.00)	32.0 (29.00, 36.00)	33.0 (29.00, 35.50)	0.8286*
Creatine phosphokinase (CPK) baseline (U/L), Median (Q1, Q3)	163.5 (75.00, 384.00)	153.0 (75.00, 373.00)	188.0 (76.00, 448.00)	0.3188^	160.5 (74.00, 375.50)	153.0 (72.00, 373.00)	175.0 (76.00, 421.00)	0.3987^
C-reactive protein (CRP) baseline (mg/l)m median (Q1, Q3)	127.0 (73.00, 201.00)	123.0 (71.00, 196.20)	140.0 (81.00, 225.10)	0.0721^	130.0 (75.00, 205.00)	123.0 (72.00, 188.00)	144.5 (86.00, 246.00)	0.0354^
Fibrinogen level baseline (gm/l), Median (Q1, Q3)	5.4 (3.99, 7.04)	5.4 (3.89, 7.02)	5.4 (4.55, 7.36)	0.2595^	5.4 (3.99, 7.23)	5.4 (3.75, 7.13)	5.4 (4.08, 7.27)	0.7919^
D-dimer level baseline, median (Q1, Q3)	1.2 (0.67, 2.62)	1.3 (0.71, 3.19)	0.9 (0.61, 1.98)	0.0007^	1.1 (0.62, 2.21)	1.1 (0.62, 2.34)	0.9 (0.62, 1.96)	0.3454^
Ferritin level baseline, median (Q1, Q3)	637.8 (348.00, 1395.00)	539.0 (301.00, 1321.00)	851.0 (425.00, 1582.00)	0.0003^	646.6 (350.00, 1319.00)	521.4 (301.00, 1193.00)	847.0 (439.45, 1487.50)	0.0008^
Blood glucose level baseline within 24 h of ICU admission, median (Q1, Q3)	10.9 (7.70, 15.30)	11.2 (7.75, 15.60)	10.1 (7.60, 14.80)	0.2785^	10.9 (7.70, 14.80)	11.0 (7.70, 14.80)	10.3 (7.70, 14.70)	0.6074^
Lowest PaO2/FiO2 ratio within 24 h of admission, Median (Q1, Q3)	80.4 (61.00, 131.50)	85.1 (62.61, 140.80)	73.2 (55.25, 103.20)	0.0001^	78.6 (58.60, 124.00)	78.3 (60.00, 131.30)	78.9 (56.60, 113.40)	0.3634^
Intensity of DVT prophylaxis, n (%) ≠								
Low intensity	62 (7.4)	52 (8.8)	10 (4.1)	0.0564^^	21 (4.7)	17 (5.7)	4 (2.7)	0.1692^^
Standard intensity	457 (54.5)	316 (53.4)	141 (57.3)		222 (49.9)	141 (47.3)	81 (55.1)	
High intensity	319 (38.1)	224 (37.8)	95 (38.6)		202 (45.4)	140 (47.0)	62 (42.2)	
Comorbidities								
Heart failure	47 (5.5)	39 (6.5)	8 (3.2)	0.0533^^	12 (2.7)	8 (2.7)	4 (2.7)	> 0.9999**
Hypertension (HTN)	469 (55.4)	334 (55.9)	135 (54.0)	0.6032^^	235 (52.6)	163 (54.7)	72 (48.3)	0.2032^^
Chronic kidney disease (CKD)	79 (9.3)	68 (11.4)	11 (4.4)	0.0014^^	20 (4.5)	14 (4.7)	6 (4.0)	0.7463^^
Diabetes mellitus (DM)	495 (58.4)	365 (61.1)	130 (52.0)	0.0138^^	255 (57.0)	176 (59.1)	79 (53.0)	0.2239^^
Dyslipidemia (DLP)	179 (21.1)	118 (19.8)	61 (24.4)	0.1318^^	94 (21.0)	59 (19.8)	35 (23.5)	0.3666^^
Chronic obstructive pulmonary disease (COPD)	17 (2.0)	14 (2.3)	3 (1.2)	0.2784^^	6 (1.3)	4 (1.3)	2 (1.3)	> 0.9999**
Asthma	71 (8.4)	50 (8.4)	21 (8.4)	0.9905^^	35 (7.8)	25 (8.4)	10 (6.7)	0.5336^^
Liver disease (any type)	19 (2.2)	17 (2.8)	2 (0.8)	0.0664^^	2 (0.4)	1 (0.3)	1 (0.7)	0.6163**

*T Test/^Wilcoxon rank sum test is used to calculate the p-value.

^^Chi square/**Fisher’s Exact teat is used to calculate p-value.

≠ Patients who received either Enoxaparin 40 mg daily or UFH 5000 Unit three times daily were grouped under the “standard dose VTE prophylaxis. Any patient who received higher than standard dose but not as treatment dose (Enoxaparin 1 mg/kg q12hr or 1.5 mg/kg q24hr or UFH infusion) was categorized as receiving “High VTE prophylaxis dose”. On the other hand, lower VTE prophylaxis considered for patient who received Enoxaparin < 40 mg/day or Unfractionated heparin (UFH) < 5000 Units three times daily/day).

### All thrombosis cases

In crude analysis, the incidence of all thrombosis cases during ICU stay was 23 (7.7%) in the control group compared to 10 (6.8%) in the tocilizumab group, p-value = 0.71. Patients who received Tocilizumab within 24 h of ICU admission had lower odds for all thrombosis cases; however, it was not statistically significant (OR 0.83; CI 0.39, 1.79; p-value = 0.63) (Table 2). On the other hand, the fibrinogen levels during ICU stay were lower in patients who received early Tocilizumab compared with the control group (beta coefficient − 0.15; CI − 0.28, − 0.02; *p*-value = 0.03); but D-dimer levels were not statistically significant between the two groups (beta coefficient 0.19; CI − 0.08, 0.47; *p*-value = 0.17) (Table2).

**Table 2:** Primary and secondary outcomes after Propensity score matching.

Outcomes	Number of outcomes/Total number of patients			Odds ratio (OR) (95%CI)	p-value $
	Control	Tocilizumab	p-value		
All thrombosis cases, n (%)	23 (7.7)	10 (6.8)	0.71^^	0.83 (0.39 ,1.79)	0.63

				Hazard ratio (HR) (95%CI)	p-value $*
30-day mortality, n (%)	99 (35.6)	26 (20.8)	0.003^^	0.57 (0.37, 0.87)	0.009
In-hospital mortality, n (%)	113 (39.2)	34 (26.6)	0.01^^	0.67 (0.46, 0.98)	0.04

				beta coefficient (Estimates) (95%CI)	p-value $**
D-dimer level (mg/l), median (Q1, Q3) ∆	3.0 (1.00, 8.06)	2.4 (1.20, 9.59)	0.89^	0.19 (− 0.08, 0.47)	0.17
Fibrinogen level, mean (SD) ∆	5.8 (2.07)	5.0 (2.42)	0.02*	− 0.15 (− 0.28, − 0.02)	0.03
Ferritin level, median (Q1, Q3) ∆	634.7 (374.1, 1582.0)	941.4 (516.7, 1701.0)	0.007^	0.17 (− 0.07, 0.40)	0.17
CRP level, median (Q1, Q3) ∆	138.0 (72.0, 240.0)	141.8 (71.0, 293.0)	0.20^	0.20 (0.00, 0.39)	0.05
Procalcitonin level, median (Q1, Q3) ∆	0.3 (0.10, 1.45)	0.3 (0.13, 1.53)	0.42^	0.20 (− 0.33, 0.72)	0.47

**^^**Chi-square test is used to calculate the p-value/** Fisher’s Exact teat is used to calculate p-value.

^Wilcoxon rank sum test is used to calculate the p-value.

^$^Logistic regression is used to calculate the OR and p-value.

^$^*Cox proportional hazards regression analysis used to calculate HR and p-value.

^$^**Generalized linear model is used to calculate estimates and p-value.

### 30-day and in-hospital mortality

After applying PS matching and at crude analysis, the 30-day and in-hospital mortality were lower in patients who received early Tocilizumab (20.8% vs. 35.6%; p-value = 0.003, and 26.6% vs. 39.2%; p-value = 0.01, respectively). Moreover, the 30-day mortality and the in-hospital mortality were statistically significantly lower in the Tocilizumab group compared with the control group at multivariable Cox proportional hazards regression analyses (Table 2).

## Discussion

Continuous efforts to find potential therapies for the management of COVID-10 suggested that TCZ might be a potential therapy for COVID-19-related hyperinflammation. This retrospective propensity score-matched study was conducted to investigate the risk of thrombotic events in critically ill patients with COVID-19. The results of the study did not find any statistically significant difference in the risk of thromboembolic events among critically ill patients with COVID-19 who were treated with TCZ in comparison to the standard of care.

Previously, Atallah et al., in a case series study noticed an increase in thrombotic events in critically ill patients with COVID-19 who had acute respiratory distress syndrome and were treated with TCZ. They reported four cases of VTE and one case of an arterial thrombotic event while being on recommended thromboprophylaxis therapy^20^. On the contrary, Sagris et al.’s did not find this increased risk of thrombosis in their meta-analysis of randomized clinical trials. In their meta-analysis, they investigated the risk of thromboembolic events in hospitalized patients with COVID-19 who received different immunomodulatory agents, including IL-6 antagonists such as tocilizumab. They found that there was no significant difference in the risk of VTE between patients with COVID-19 who received immunomodulatory agents and those who received standard care (OR 0.52, 95%CI 0.22–1.20; I^2^ = 6%)^22^. In addition, Viswanatha et al.^21^ in a meta-analysis that included 5686 patients with COVID-19 has shown a non-significant difference in the occurrence of pulmonary embolism during the TCZ therapy compared to the standard of care (OR 1.01, 95%CI 0.45–2.26, I^2^ = 0%). Likewise, our analysis did not find any significant increase in the risk of thrombosis for patients with COVID-19 who were treated with TCZ.

A single retrospective cohort study by Campochiaro et al., evaluated 65 patients with a median PaO_2_:FiO_2_ ratio at baseline of 107 (82–181) in the tocilizumab group and 124 (91–172) in the control group. They found that the higher median PaO_2_:FiO_2_ ratio at baseline to be a predictor for clinical improvement at day 28 in the multivariable analysis. However, there was no statistical difference in the rate of pulmonary thrombosis cases between patients in the tocilizumab group compared to patients in the control group in the study^14^. Most of these patients presented with a median of PaO_2_:FiO_2_ ratio above 100, while in our cohort the lowest median PaO_2_:FiO_2_ ratio within 24 h of admission was 78.6 (58.6–124.0) with a mean SOFA and APACHE II scores of 4 and 12 respectively. Although we had more patients with severely impaired oxygenation at baseline, we still observed the mortality benefit for the use of TCZ, yet further investigations are needed to address the impact of higher SOFA and APACHE II score on patients’ outcomes.

Besides that, some of the literature implied that the risk of TCZ-induced thrombosis could be serious. Chan et al. in a retrospective cohort study that included 24 patients with COVID-19 who were treated with TCZ reported a tendency for increased thromboembolism-related mortality. However, in their study they included both patients who were on anticoagulation for thromboprophylaxis and those who were not on any thromboprophylaxis while being on TCZ therapy. Moreover, they only reported one case of death out of 17 treated with TCZ while being on thromboprophylaxis^17^. In addition, Kimming et al., compared the risk of death in critically ill patients with COVID-19 when treated with TCZ or the standard of care. In contrast to our findings, they reported higher mortality in the TCZ group compared to the standard of care (35.2 vs. 19.3%; *p* = 0.020). However, Kimming et al.^16^ included five post-transplantation patients in the TCZ group versus none in the control group (9.5% vs. 0%; *p* = 0.025), and more patients were on other immunosuppressive agents in the TCZ compared to the other group (13.0% vs. 1.8%; *p* = 0.029).

While the suggested risk was based on small observational studies with major limitations in the design of these studies, there were some studies that indicated the survival benefits for TCZ. The RECOVERY trial indicated that TCZ was associated with a lower risk of death in hospitalized patients with COVID-19. The study also demonstrated that the use of TCZ reduced the need for mechanical ventilation and the length of stay before being discharged alive from hospital. Mortality as an objectively measured outcome most probably would not be affected by the fact that the RECOVERY did not blind patients or investigators during the trial. While outcomes that were more subjectively measured, such as disease activity, were thought to be susceptible to bias^23^. Moreover, the Viswanatha et al.^21^ meta-analysis found that TCZ therapy was associated with a lower risk of mortality compared to the standard of care (OR − 0.11, 95%CI − 0.18 to − 0.04), but with significant heterogeneity among studies (I^2^ = 88%, *p* = 0.001). Similarly, our study revealed that the use of TCZ was associated with a lower risk of mortality in critically ill patients when compared to the standard care; patients treated with TCZ had a 43% lower risk of 30-day mortality and a 33% lower risk of in-hospital mortality.

In addition, the current literature reports conflicting results about the impact of TCZ on inflammatory markers in patients with COVID-19 in critical care. Sami Ullah et al., conducted an open-label, randomized clinical trial to investigate the impact of TCZ in patients with COVID-19 while focusing on the remission of the cytokine release syndrome. The study did not reveal any statistically significant difference between the TCZ and control group in inflammatory markers (CRP, ferritin, D-dimer or LDH) while the coagulation parameters showed a significant difference in the median change from baseline between the TCZ and control group (INR 0.12 vs. − 0.07; *p* ≤ 0.001; aPTT 0.42 vs. − 1.16; *p* ≤ 0.001; prothrombin time (PT) 0.31 vs. − 0.96; *p* ≤ 0.001; and platelet count − 12.34 vs. − 1.47; *p* = 0.012)^24^. In the Atallah et al.^20^ case series, they observed a decline in inflammatory markers, such as D-dimer and fibrinogen, in hospitalized patients with COVID-19 while being on TCZ therapy. While Chan et al.^17^ observed a transient elevation in D-dimer during the TCZ therapy. In comparison to the previous studies, the peak D-dimer levels did not significantly change when patients received TCZ in our study (*ꞵ* 0.19; 95%CI − 0.08 to 0.47), while there was a significant reduction in the fibrinogen levels when these patients were receiving care in ICU (*ꞵ* − 0.15; 95%CI − 0.28 to − 0.02). Since it is not significant in our study, it is possible that the impact of the TCZ on the coagulation profile (INR, aPTT, and PT) caused the previously observed risk of thrombosis.

The results of this study add to the available evidence about TCZ, but it has some limitations that need to be taken into consideration. The study’s retrospective nature and the restrictions posed by COVID-19 on access to invasive diagnostic techniques at the onset of the pandemic may have limited the accurate capture of the number of thrombosis events. While the majority of baseline characteristics were similar between the groups, some were slightly different; thus, the propensity score matching technique was used in an effort to minimize the impact of these characteristics and other unmeasured confounders on the evaluated outcomes. Additionally, this study included clear reports on the antithrombotic treatment modalities used in these patients and classified them according to the standard VTE prophylaxis doses. In comparison to other previous studies, we focused our investigation on critically ill patients with COVID-19 who were on thromboprophylaxis and treated with/without TCZ. Our findings may help healthcare practitioners to decide on using TCZ in critically ill patients with COVID-19 who are adequately managed with anticoagulation for thromboprophylaxis and encourage their risk and benefit balanced decision.

## Conclusion

The use of tocilizumab in critically ill patients with COVID-19 was not associated with higher thrombosis events or peak D-dimer levels. On the other hand, fibrinogen levels, 30-day and in-hospital mortality were significantly lower in the tocilizumab group. Further randomized controlled trials are needed to confirm our findings.

## Data Availability

The datasets supporting the conclusions of this article are available from the corresponding author on reasonable request.

## References

[1] World Health Organization. WHO Coronavirus Disease (COVID-19) dashboard with vaccination data|WHO Coronavirus (COVID-19) dashboard with vaccination data [Internet]. World Health Organization 1–5. https://covid19.who.int/%0Ahttps://covid19.who.int/%0Ahttps://covid19.who.int/region/searo/country/bd (2021)

[2] Kutsuna S (2021). Clinical manifestations of coronavirus disease 2019. JMA J..

[3] Huang C, Wang Y, Li X, Ren L, Zhao J, Hu Y (2020). Clinical features of patients infected with 2019 novel coronavirus in Wuhan, China. Lancet.

[4] Vardhana SA, Wolchok JD (2020). The many faces of the anti-COVID immune response. J. Exp. Med..

[5] Morris BJ, Alazawi W, Kanoni S (2021). Interleukin-6 receptor antagonists in critically ill patients with Covid-19. N. Engl. J. Med..

[6] Zhao J, Cui W, Tian BP (2020). Efficacy of tocilizumab treatment in severely ill COVID-19 patients. Crit. Care.

[7] Yu SY, Koh DH, Choi M, Ryoo S, Huh K, Yeom JS (2022). Clinical efficacy and safety of interleukin-6 receptor antagonists (tocilizumab and sarilumab) in patients with COVID-19: A systematic review and meta-analysis. Emerg. Microbes Infect..

[8] Shankar-Hari M, Vale CL, Godolphin PJ, Fisher D, Higgins JPT, Spiga F (2021). Association between administration of IL-6 antagonists and mortality among patients hospitalized for COVID-19: A meta-analysis. JAMA J. Am. Med. Assoc..

[9] COVID-19 Treatment Guidelines Panel. Coronavirus Disease 2019 (COVID-19) Treatment Guidelines. National Institutes of Health. Available at https://www.covid19treatmentguidelines.nih.gov/ Accessed [15/07/2023] 34003615

[10] Lamontagne F, Agoritsas T, MacDonald H, Leo YS, Diaz J, Agarwal A (2020). A living WHO guideline on drugs for covid-19. BMJ.

[11] Hafez W, Ziade MA, Arya A, Saleh H, Abdelshakor M, Alla OF (2022). Treatment outcomes of tocilizumab in critically-Ill COVID-19 patients, single-centre retrospective study. Antibiotics.

[12] Guaraldi G, Meschiari M, Cozzi-Lepri A, Milic J, Tonelli R, Menozzi M (2020). Tocilizumab in patients with severe COVID-19: A retrospective cohort study. Lancet Rheumatol..

[13] Rosas IO, Bräu N, Waters M, Go RC, Hunter BD, Bhagani S (2021). Tocilizumab in hospitalized patients with severe Covid-19 pneumonia. N. Engl. J. Med..

[14] Campochiaro C, Della-Torre E, Cavalli G, De Luca G, Ripa M, Boffini N (2020). Efficacy and safety of tocilizumab in severe COVID-19 patients: A single-centre retrospective cohort study. Eur. J. Intern. Med..

[15] Al Sulaiman K, Aljuhani O, Bin Salah K, Korayem GB, Eljaaly K, Al Essa M (2021). Single versus multiple doses of Tocilizumab in critically ill patients with coronavirus disease 2019 (COVID-19): A two-center, retrospective cohort study. J. Crit. Care.

[16] Kimmig LM, Wu D, Gold M, Pettit NN, Pitrak D, Mueller J (2020). IL-6 inhibition in critically Ill COVID-19 patients is associated with increased secondary infections. Front. Med..

[17] Chan KH, Patel B, Podel B, Szablea ME, Shaaban HS, Guron G (2021). Tocilizumab and thromboembolism in COVID-19: A retrospective hospital-based cohort analysis. Cureus.

[18] Stone RL, Nick AM, McNeish IA, Balkwill F, Han HD, Bottsford-Miller J (2012). Paraneoplastic thrombocytosis in ovarian cancer. N. Engl. J. Med..

[19] Zhang Y, Zhang Z, Wei R, Miao X, Sun S, Liang G (2020). IL (interleukin)-6 contributes to deep vein thrombosis and is negatively regulated by miR-338-5p. Arterioscler. Thromb. Vasc. Biol..

[20] Atallah B, El Nekidy W, Mallah SI, Cherfan A, Abdelwareth L, Mallat J (2020). Thrombotic events following tocilizumab therapy in critically ill COVID-19 patients: A Façade for prognostic markers. Thromb. J..

[21] Viswanatha GL, Male CHK, Shylaja H (2022). Efficacy and safety of tocilizumab in the management of COVID-19: A systematic review and meta-analysis of observational studies. Clin. Exp. Rheumatol..

[22] Sagris D, Florentin M, Tasoudis P, Korompoki E, Gatselis N, Giamarellos-Bourboulis EJ (2021). Immunomodulation and reduction of thromboembolic risk in hospitalized COVID-19 patients: Systematic review and meta-analysis of randomized trials. J. Clin. Med..

[23] Horby PW, Mafham M, Bell JL, Linsell L, Staplin N, Emberson J (2020). Lopinavir–ritonavir in patients admitted to hospital with COVID-19 (RECOVERY): A randomised, controlled, open-label, platform trial. Lancet.

[24] Ullah S, Abid R, Haider S, Khuda F, Albadrani GM, Abdulhakim JA (2022). Assessment of tocilizumab (humanized monoclonal antibody) for therapeutic efficacy and clinical safety in patients with coronavirus disease (COVID-19). Medicina.

[25] Al-Sulaiman KA (2021). Clinical features and outcomes of critically ill patients with coronavirus disease 2019 (COVID-19): A multicenter cohort study. Int. J. Infect. Dis..

[26] Mouli TC, Patnaik RK, Mishra SB (2023). Tocilizumab in severe COVID-19 pneumonia: A retrospective case-control study from eastern India. Indian J. Anaesthes..

